# Clinical and genomic analysis of virulence-related genes in bloodstream infections caused by *Acinetobacter baumannii*

**DOI:** 10.1080/21505594.2022.2132053

**Published:** 2022-10-28

**Authors:** Bing Bai, Brianna M. Eales, Wei Huang, Kimberly R. Ledesma, Paul R. Merlau, Guiqiu Li, Zhijian Yu, Vincent H. Tam

**Affiliations:** a Department of Infectious Diseases and Shenzhen Key Laboratory for Endogenous Infections, The 6th Affiliated Hospital of Shenzhen University Health Center, Shenzhen, Guangdong, China; b Department of Pharmacy Practice and Translational Research, University of Houston, Houston, Texas, USA; c Bacteriology & Antibacterial Resistance Surveillance Laboratory, Shenzhen Institute of Respiratory Diseases, Shenzhen People’s Hospital (The Second Clinical Medical College, Jinan University, The First Affiliated Hospital, Southern University of Science and Technology), Shenzhen, Guangdong, China

**Keywords:** Gram-negative bacteria, bacteraemia, whole genome sequencing, outcome

## Abstract

*Acinetobacter baumannii* has emerged as a common cause of bloodstream infections, which is associated with high mortality and long periods of hospitalization. To advance the medical care of our patients, the study was designed to identify microbial characteristics associated with poor clinical outcomes. A collection of 32 *A. baumannii* bloodstream isolates with diverse genetic backgrounds (as determined by multilocus sequence typing) was studied. These isolates were recovered by unique patients (18 males, 14 females; age range: 17 days to 87 years) between 2011 and 2018. A sequential screening approach (cross-referencing analyses using different endpoints) was used to identify isolates with the best correlation between bacterial virulence and clinical prognosis. Isolates associated with more rapid *in vitro* growth rate, shorter median survival time in pre-clinical infection models, and hospital mortality were selected as candidates for high virulence, while those with opposite characteristics were selected as controls with low virulence. Whole genome sequencing was undertaken in the most promising clinical isolates. We found five virulence genes (beta-hemolysin/cytolysin, Cpi-1a + Cpi-1 (SPI-1 like), enhanced entry proteins, FbpABC, Paa) and 1 secretory system (T6SS) only present in a highly virulent isolate (AB23), compared to a low virulence control isolate (AB6). These genetic elements could be associated with the poor prognosis of *A. baumannii* bacteraemia and further investigations are warranted.

## Introduction

*Acinetobacter baumannii* is an aerobic and non-fermentative Gram-negative bacillus, commonly implicated in resistance to multiple antibiotics [1]. It has been estimated that approximately 1,000,000 people are infected with *A. baumannii* every year worldwide [1,2]. *A. baumannii* often causes serious infections, such as hospital-acquired pneumonia, skin/soft tissue infections, urinary tract infections, and secondary meningitis [3–5]. Bloodstream infections in critically ill patients due to *A. baumannii* are often associated with a high mortality, and contribute to a prolonged hospital stay, as well as high treatment costs [6,7]. The crude mortality rates of *A. baumannii* bacteraemia have been reported to be between 30 and 76% [7–10].

Identification of risk factors associated with mortality in *A. baumannii* bloodstream infections is crucial for early implementation of appropriate therapy and improving patient outcome. From previous studies, risk factors identified to be associated with worse prognosis included immunosuppression [8–10], drug resistance [11], severity of underlying illness [12–14], inappropriate antimicrobial therapy [15], prior antibiotic exposure [10,12,13], Pitt bacteraemia score, and Acute Physiology and Chronic Health Evaluation (APACHE) II score at the onset of infection [16]. Many of these acute host abnormalities represent manifestations of the late stages in the disease course. Although predictions are reasonably accurate, medical interventions to improve patient outcomes are often too late to be effective. To advance medical care of our patients, this study aims to identify microbial characteristics associated with poor clinical outcomes. These *in vitro* and *in vivo* findings could improve patient prognosis at an early stage of infection, in order to facilitate timely and effective interventions.

## Materials and methods

### Bacteria

Bloodstream isolates of *A. baumannii* were retrieved from Shenzhen Nanshan People’s Hospital and the Fourth Affiliated Hospital of Harbin Medical University (Shenzhen, China) between 2011 and 2018. The isolates were characterised using a VITEK®2 Compact Bacterial Identification System (BioMérieux, Marcy l’Etoile, France) and validated by MALDI-TOF mass spectrometry. Antimicrobial susceptibility testing of the isolates was performed using the VITEK 2 system (BioMérieux, Marcy l’Etoile, France) and interpreted based on CLSI criteria. The clonal relatedness of the isolates was assessed using multilocus sequence typing (MLST). The Oxford scheme developed by Bartual and co-workers was designed to identify the following seven internal house-keeping genes: citrate synthase (gltA), DNA gyrase subunit B (gyrB), glucose dehydrogenase B (gdhB), homologous recombination factor (recA), 60-kDa chaperonin (cpn60), glucose-6-phosphate isomerase (gpi), and RNA polymerase sigma factor (rpoD) [17]. Allele numbers and sequence types (STs) were assigned in accordance with the MLST database (http://www.mlst.com/). The isolates were stored at −80ºC and sub-cultured on 5% blood agar (Hardy Diagnostics, Santa Maria, CA, USA) at least twice prior to downstream investigations.

### Clinical outcomes

To enhance the clinical relevance of the isolate screening process, pertinent demographic characteristics of the patient population (i.e. age, gender, source of bacteraemia) and all-cause hospital mortality were assessed from electronic medical records.

### Biofitness

The *in vitro* growth rate of the isolates was assessed using the BacterioScan 216Dx instrument, as described previously [18]. Bacterial inocula were prepared by inoculating an overnight culture in Mueller-Hinton broth. When log-growth phase was achieved, bacterial density was estimated using absorbance at 630 nm. The suspension was diluted accordingly to achieve a baseline inoculum of approximately 1–5 × 10^5^ CFU/ml, and an optical signal was obtained from a 2-ml aliquot every 5 min for 6 h. The equivalent colony counts were enumerated based on a correlation to viable bacterial burden from pilot experiments. The *in vitro* growth rates were estimated by fitting a non-linear model (exponential growth with saturation) to the growth profiles, as shown previously [19]. The growth rates of different isolates were ranked in a descending order and correlated to the hospital outcomes of the patients. We postulate that biofitness is associated with bacterial virulence. Therefore, fast growing (above median growth rate) bacteria associated with hospital mortality were selected for *in vivo* investigations. In contrast, slow growing (below median growth rate) bacteria not associated with hospital mortality were selected as controls.

### Galleria mellonella killing assay

The virulence of selected bacterial isolates was initially screened using a modified *Galleria mellonella* (wax moth) model [20–22]. For each bacterial isolate, 10 randomly selected larvae (weight ranging 200–300 mg) were injected with 10 μl of an exponentially growing *A. baumannii* (1–5 × 10^6^ CFU/ml in saline), via the larval haemolymph behind the last proleg. The density of the inoculum was confirmed by quantitative culture. As a negative control, 10 μl of sterile saline was injected into the same number of larvae to rule out mortality due to trauma. The larvae were kept in a humidified incubator at 37°C, and their survival was checked every hour for up to 48 h. The median survival time of larvae infected with different isolates was correlated to hospital mortality.

### Virulence in an in vivo infection model

The virulence of selected *A. baumannii* isolates was further validated using a neutropenic murine pneumonia model [23]. Eight to ten weeks old female Swiss Webster mice (weight 22–25 g) obtained from Envigo (Indianapolis, IN, USA) were used in all experiments. The animals received food and water *ad libitum*. The animals were rendered neutropenic by 2 doses of cyclophosphamide administered intraperitoneally (150 mg/kg on day −4, and 100 mg/kg on day −1). For infection, mice (anesthetized with intraperitoneal ketamine/xylazine) were inoculated with approximately 1 × 10^8^ CFU (in 10 ul of saline) via the trachea under larynogoscopic guidance. Three mice were sacrificed to verify the bacterial burden in the lungs at baseline. Infected mice (n = 5 per isolate) were monitored every 8 h for up to 4 days. At each observation, animals found moribund were euthanized (by CO_2_ asphyxiation) and were reckoned as death observed at the following time point. All surviving mice at the end of the experiment were sacrificed. Bacterial burden in the lungs of each animal was determined using quantitative culture to ascertain the baseline inoculum and the cause of death. The onset of mortality was compared using Kaplan – Meier survival analysis and log-rank test (Prism version 5.02, GraphPad Software, Inc., San Diego, CA, USA). Right censoring was used if the mortality endpoint was not directly observed.

### Genomic characterisation

To identify genetic determinant(s) associated with bacterial virulence, whole genome sequencing was undertaken in the most promising clinical isolates. Bacteria were cultured in Luria – Bertani (LB) broth with shaking overnight at 37°C. Overnight cultures were diluted 1/100 and re-cultured until the absorbance at 600 nm was 0.6–0.8. The SDS method was used to extract genomic DNA from each isolate. Bacterial genome sequencing was performed using Illumina HiSeq 2500 platform (Illumina, San Diego, CA, USA). Furthermore, *fastp* was used to remove low-quality and low-complexity reads, and polyG/polyX tails [24]. The genomes were consequently assembled with de novo SPAdes Genome Assembler (version 3.12.0) [25]. To predict gene functions, a whole genome Blast search (E-value less than 1e-5, minimal alignment length percentage larger than 40%) was performed. Potential virulence factor(s) were identified by cross-referencing Virulence Factor Database (VFDB) using the ABRicate program (version 0.8.7) [26]. Core genes and specific genes were analysed by the CD-HIT rapid clustering of similar proteins software with a threshold of 50% pairwise identity and 0.7 length difference cut-off in amino acid. A Venn figure was used to show the relationships among the sequences.

## Results

### Bacteria, clinical outcomes, and in vitro growth rates

A collection of bloodstream isolates obtained from 32 unique patients (male: 18, female: 14; age range: 17 days to 87 years) were included in the study. Only a single isolate was obtained from each patient. A significant proportion of the isolates were recovered from patients in intensive care units (ICU) (43.8%). The primary source of bacteraemia was the respiratory tract (75%). A total of 5 different sequence types were identified in 21 isolates from the *A. baumannii* MLST database (using the Oxford scheme). The predominant sequence types were ST195 and ST457, accounting for 25.0% (8 isolates) and 21.9% (7 isolates), respectively. Most isolates of *A. baumannii* were resistant to at least three antimicrobial classes (e.g. beta-lactams, aminoglycosides, fluoroquinolones, tetracyclines). Hospital (all-cause) mortality rate was 40.6%. Overall model fits to the *in vitro* growth profiles of the isolates were satisfactory (r^2^ > 0.985). The best-fit growth rate ranged from 1.193 h^−1^ to 2.132 h^−1^.

### Galleria mellonella killing assay

Sixteen isolates were investigated. Among these, the growth rate was above median in 10 isolates. At 48 h post-infection, the mortality of larvae infected with different isolates ranged from 0% to 100%. In comparison, the mortality of larvae injected with saline was ≤10%. Isolates with a median survival time of less than 24 h (n = 9) were associated with a higher proportion of hospital mortality (55.6% vs. 0%, p = 0.034 by Fisher exact test). Five isolates were deemed to be the most relevant; their pertinent characteristics are as shown in Table 1.

**Table 1.** Pertinent isolate characteristics.

Isolate	Patients’ gender	Patients’ age (year)	Source of bacteraemia	Antimicrobial phenotype	Hospital outcome	MLST	Growth rate (h^−1^, 95% CI)	*Median survival (h)	Remarks
AB6	F	16	Wound	MDR	Alive	NT	1.779 (1.752–1.806)	>48	Control
AB8	M	72	Unknown	Non-MDR	Alive	457	1.547 (1.512–1.582)	>48	Control
AB23	M	74	Lung	Non-MDR	Expired	NT	1.860 (1.831–1.890)	6	-
AB26	F	67	Lung	MDR	Expired	NT	1.990 (1.953–2.026)	7	-
AB31	M	66	Lung	MDR	Alive	208	1.627 (1.600–1.653)	>24	Control

F-female; *M*-male; MDR – multidrug resistance, as nonsusceptibility to ≥ 1 agent in ≥ 3 antimicrobial categories; MLST – multilocus sequence typing; NT – Non-typable.

*In *Galleria mellonella* model (n = 10).

### Virulence in an in vivo infection model

Out of the five isolates shortlisted, two isolates (AB6 and AB23, deemed to be the most different) were further examined in the neutropenic murine pneumonia model. Overall mortality was 100% at 96 h post-infection for both isolates. However, the median survival times were found to be 80 h (for AB6) and 64 h (for AB23), respectively (p = 0.043). The bacterial burden in the lungs of all succumbed animals was significantly higher than those observed at baseline (2 h post-infection), supporting that pneumonia was the most likely cause of death (data not shown).

### Genomic characterization

**Figure 1.**
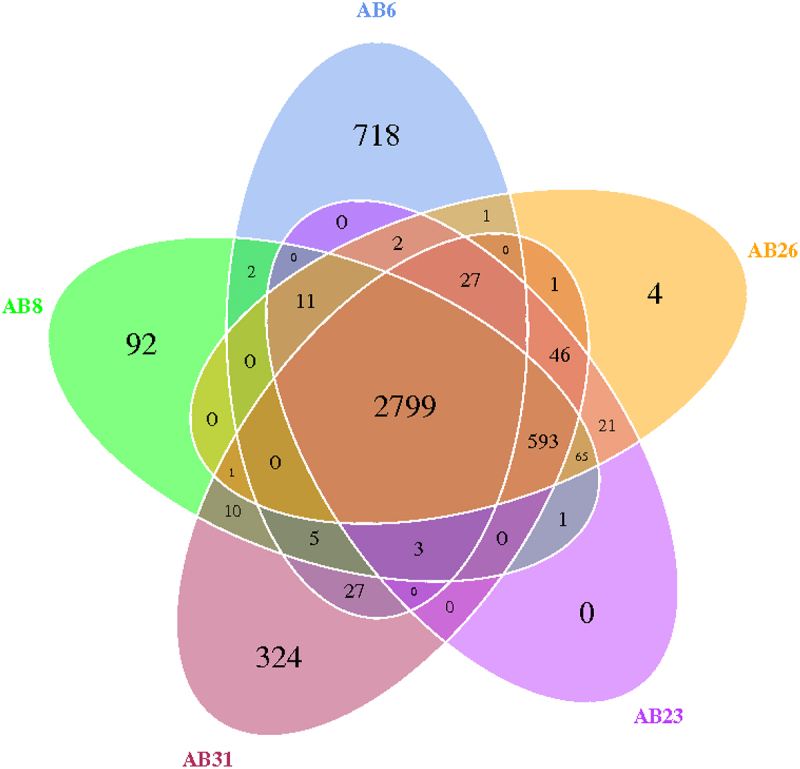
Venn diagram of different genes detected in all 5 isolates.

To further investigate the molecular and genomic features of bloodstream *A. baumannii* isolates, whole genome sequencing and analysis were conducted for 5 *A. baumannii* isolates (Table 2). A total of 4753 pan genes were detected, including 2799 core genes and 1954 dispensable genes. As shown in Figure 1, isolate AB6 showed significant differences from others, especially isolate AB23. A total of 378 virulence genes have been identified from the VFDB. Comparative analysis identified five virulence genes (beta-hemolysin/cytolysin, Cpi-1a + Cpi-1 (SPI-1 like), enhanced entry proteins, FbpABC, Paa) and a type VI secretory system (T6SS) that were uniquely present in isolate AB23 but not in isolate AB6 (Table 3). The genome sequences of *A. baumannii* AB6, AB8, AB23, AB26, and AB31 were deposited in GenBank (accession numbers: JAOAOM000000000, JAOAON000000000, JAOANQ000000000, JAOAOO000000000, and JAOBQI000000000, respectively).

Pan genes describe the union of gene sequence entities that exist in all five isolates; core genes describe the gene sequences shared by all isolates. After the core genes are removed from the pan genes, the dispensable genes are obtained. Specific genes describe the gene sequences existing only within a particular isolate.

**Table 2.** Quality of DNA sequencing and genome information of *A. baumannii* isolates examined.

Isolate	Total Num (>500bp)	N50 Length (bp)	N90 Length (bp)	Sequence GC%	Genome size (bp)	Gene number (#)	Gene total length (bp)	Gene average length (bp)	Gene length/Genome (%)
AB6	145	54,969	15,612	39	3,844,440	3,716	3,314,676	892	86.2
AB8	53	147,699	42,862	38.95	3,880,200	3,714	3,403,065	916	87.7
AB23	56	168,007	42,864	38.95	3,918,936	3,720	3,415,293	918	87.2
AB26	60	158,175	42,707	38.95	3,923,171	3,724	3,424,644	920	87.3
AB31	57	143,736	50,558	39.09	4,148,595	4,004	3,614,154	903	87.1

**Table 3.** Potential genes associated with virulence identified.

VFDB internal ID	VF ID	VF name	Related genes
VFG005770	CVF171	Beta-hemolysin/cytolysin	acpC acyl carrier protein AcpC
VFG041842	SS016	Cpi-1a + Cpi-1 (SPI-1 like)	armR two-component response regulator
VFG010518	CVF348	Enhanced entry proteins	lidL TPR repeat protein
VFG001206	VF0272	FbpABC	fbpC ironIII ABC transporter, ATP-binding protein
VFG000839	VF0194	Paa	paa outer membrane adhesin Paa
VFG041142	SS193	T6SS	tssL/bscP putative type VI secretion system protein TssL
VFG041143	SS193	T6SS	tssK/bscO putative type VI secretion system protein TssK
VFG041146	SS193	T6SS	tssB/bscL putative type VI secretion system protein TssB
VFG041147	SS193	T6SS	tssC/bscK putative type VI secretion system protein TssC
VFG041148	SS193	T6SS	tssD/hcp/bscJ putative type VI secretion system protein TssD
VFG041149	SS193	T6SS	tssE/bscI putative type VI secretion system protein TssE
VFG041152	SS193	T6SS	tssH/clpV/bscF putative type VI secretion system protein TssH
VFG041154	SS193	T6SS	bscD putative outer membrane protein
VFG041156	SS193	T6SS	tssM/icmF/bscB putative type VI secretion system protein TssM
VFG041157	SS193	T6SS	bscA subfamily M15C metalopeptidase

VF – virulence factor; ID – identification.

## Discussion

*A. baumannii* is emerging as a pathogen of great concern because it is associated with high mortality, and infections due to *A. baumannii* are difficult to treat due to high levels of antibiotic resistance. The World Health Organization (WHO) has recently identified carbapenem-resistant *A. baumannii* as one of the critical-priority bacteria, which new and effective antibiotic development are urgently required [27]. The clinical impact of nosocomial *A. baumannii* infections has been a matter of continuing debate. The clinical outcome of each patient could be attributed to a combination of many factors. Although many studies reported high overall mortality rates in patients with *A. baumannii* bacteraemia [28], relatively little is known regarding the molecular basis of pathogenesis.

The role of several virulence-related factors, such as proteases, phospholipases, outer membrane porins, lipopolysaccharides (LPS), capsular polysaccharides, iron-chelating systems, and protein secretion systems have been previously investigated [29]. Using a similar *G. mellonella* model, approximately 300 genes in *A. baumannii* were thought to be necessary for survival and/or growth [30]. The investigators speculated that these genes might be related to nutrient acquisition and/or resistance to the *G. mellonella* immune responses. Similar genes (e.g. those involved in zinc/iron acquisition, capsule/LPS biosynthesis, amino acid metabolism, and acquisition) were also identified by another investigator group using a murine pneumonia model [31].

**Figure 2.**
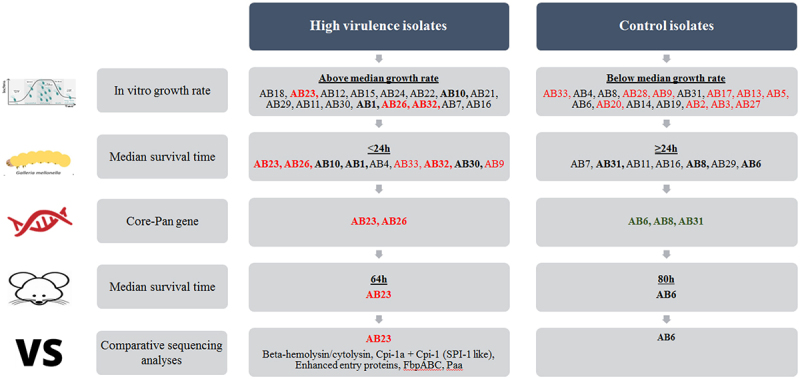
Schematic illustration of the study processes.

In this study, 32 clinical *A. baumannii* bloodstream isolates with diverse genetic backgrounds were investigated. It was noteworthy that some patients with *A. baumannii* bloodstream infection expired despite receiving timely anti-infective treatment (i.e. within 48 h of index culture). However, others had favourable outcomes even when effective anti-infective therapy was delayed (i.e. initiated beyond 48 h of index culture). In view of the complex and heterogeneous patients’ clinical backgrounds, our study design would not allow us to attribute patient mortality simply to bacterial virulence, antibiotic resistance, or other non-infectious disease causes. From a limited collection of clinical isolates, a sequential screening approach was used to shortlist the most relevant isolates for downstream prioritisation. We cross referenced different analyses in clinical outcome, *in vitro* bacterial growth rate, and survival time in infected animal (Figure 2). Whole genome sequencing was further undertaken in a focused collection of isolates to reduce irrelevant noise in our evaluation of different genes. Among the five isolates, isolate AB6 had the most specific genes (718 specific genes). Pairwise comparison indicated that AB6 and AB23 had the most differential genes, leading us to believe that these two isolates were the most different.

The genome of the five isolates encoded 378 virulence genes. Primary virulence classes including offensive (adherence and invasion, induction of apoptosis, killing of competing bacteria, neutrophil influx), defensive (biofilm formation, persistence, growth in serum, survival in tissue infection, evasion of the host immune response, in vivo survival, host colonization), regulation of virulence-associated genes and non-specific virulence factors were identified. Comparative sequencing analyses led to the identification of five virulence genes and one secretory system in a highly virulent isolate (AB23), but not in a low virulence control isolate (AB6). One of the virulence genes identified was the phenylacetic acid catabolism pathway (encoded by the paa system), which was previously reported to enable *A. baumannii* to evade neutrophil chemotaxis [2,32]. In addition, type VI secretion system (T6SS) is widespread among Gram-negative bacteria. It can be used to target proteins against other bacteria and eukaryotic cells [33], allowing the bacteria to colonize, disseminate, and evade host innate immune responses. Besides paa and T6SS, little is known for other virulence genes identified in this study in *A. baumannii* infections. However, these genes have been shown to be involved in virulence in other bacterial species [34–37], supporting the hypothesis that these genes could be potential virulence genes in *A. baumannii* infections.

There are several limitations in our study. Our clinical specimens came from two hospitals within a geographic region, which could limit the generalisation of our results. We had a relatively small sample size; therefore, we might not have been able to examine the contribution of all possible risk factors. Histopathology and chemokines data were not available, they could have further strengthened our findings in addition to the mortality difference observed in infected mice. Finally, we did not verify the impact contributed by specific genes (or gene clusters) identified in our screening. Nonetheless, we believe our preliminary findings are of value guiding future investigations to advance care for our patients.

## Conclusions

Unique virulence genes and a secretory system were identified in a highly virulent isolate of *A. baumannii*, using a sequential screening approach with cross-validation in different pre-clinical infection models. These genetic elements could be associated with the poor prognosis of *A. baumannii* bacteraemia and further investigations are warranted.

## Data Availability

The data that support the findings of this study are openly available in National Center for Biotechnology Information (NCBI) at https://www.ncbi.nlm.nih.gov/, accession numbers: JAOAOM000000000, JAOAON000000000, JAOANQ000000000, JAOAOO000000000, and JAOBQI000000000, respectively.
